# MicroRNA-145 targets MUC13 and suppresses growth and invasion of pancreatic cancer

**DOI:** 10.18632/oncotarget.2281

**Published:** 2014-07-30

**Authors:** Sheema Khan, Mara C. Ebeling, Mohd S. Zaman, Mohammed Sikander, Murali M. Yallapu, Neeraj Chauhan, Ashley M. Yacoubian, Stephen W. Behrman, Nadeem Zafar, Deepak Kumar, Paul A. Thompson, Meena Jaggi, Subhash C. Chauhan

**Affiliations:** ^1^ Department of Pharmaceutical Sciences and Center for Cancer Research, University of Tennessee Health Science Center, Memphis, Tennessee, USA; ^2^ Cancer Biology Research Center, Sanford Research/USD, Sioux Falls, South Dakota, USA; ^3^ Department of Pathology, University of Tennessee Health Science Center, Memphis, Tennessee, USA; ^4^ Department of Surgery, University of Tennessee Health Science Center, Memphis, Tennessee, USA; ^5^ Department of Biological and Environmental Sciences, University of the District of Columbia, Washington, District of Columbia; ^6^ Methodology and Data Analysis Center, Sanford Research, Sioux Falls, South Dakota, USA

**Keywords:** Pancreatic cancer, MUC13, MicroRNA, Tumor suppressor, Diagnostics, Therapeutics

## Abstract

Pancreatic cancer has a poor prognosis due to late diagnosis and ineffective therapeutic multimodality. MUC13, a transmembrane mucin is highly involved in pancreatic cancer progression. Thus, understanding its regulatory molecular mechanisms may offer new avenue of therapy for prevention/treatment of pancreatic cancer. Herein, we report a novel microRNA (miR-145)-mediated mechanism regulating aberrant MUC13 expression in pancreatic cancer. We report that miR-145 expression inversely correlates with MUC13 expression in pancreatic cancer cells and human tumor tissues. miR-145 is predominantly present in normal pancreatic tissues and early Pancreatic Ductal Adenocarcinoma (PDAC) precursor lesions (PanIN I) and is progressively suppressed over the course of development from PanIN II/III to late stage poorly differentiated PDAC. We demonstrate that miR-145 targets 3′ untranslated region of MUC13 and thus downregulates MUC13 protein expression in cells. Interestingly, transfection of miR-145 inhibits cell proliferation, invasion and enhances gemcitabine sensitivity. It causes reduction of HER2, P-AKT, PAK1 and an increase in p53. Similar results were found when MUC13 was specifically inhibited by shRNA directed at MUC13. Additionally, intratumoral injections of miR-145 in xenograft mice inhibited tumor growth *via* suppression of MUC13 and its downstream target, HER2. These results suggest miR-145 as a novel regulator of MUC13 in pancreatic cancer.

## INTRODUCTION

Pancreatic cancer (PanCa) is the fourth leading cause of cancer related death in the United States, with a 5-year survival rate of less than 5% [1]. The development of an effective treatment requires further investigations of the molecular mechanisms that underlie its aggressive nature. MicroRNAs (miRNAs) are small, noncoding RNAs that are highly associated with cancer initiation and progression. Disruption of miRNAs has important implications in etiology, treatment and pathogenesis of cancer, including PanCa [2-4]. They bind to partially complementary sequences in mRNAs, targeting them for degradation and/or inhibiting their translation, thereby, downregulating protein expression [5, 6]. Important advances have taken place in understanding the molecular progression of PanCa and several important targets have been identified and experimentally tested for their functional participation in the disease process [7-9]. We have recently reported a role of mucin 13 (MUC13) in PanCa that suggests its potential use as a diagnostic and therapeutic target in PanCa [10]. The MUC13 mucin is aberrantly overexpressed in PanCa and the exogenous expression of MUC13 augments tumorigenic features such as enhanced cell proliferation, cell motility, cell invasion, and *in vivo* tumor growth [10]. Additionally, it has been demonstrated that the expression of MUC13 correlates with the expression/activation of key oncogenes, *HER2, PAK1, ERK, Akt,* and *S100A4,* and the decreased expression of p53, a tumor suppressor [10].

The present work suggests that miR-145 is a tumor suppressor in pancreatic cancer and a novel regulator of MUC13 expression. Recent studies showed that miR-145 targets ADAM17 and suppresses cell invasion in hepatocellular [11] and head and neck cancers [12]. Moreover, miR-145 overexpression directly targets AKT-3 in thyroid cancer [13]. It has also been demonstrated that miR-145 targets MUC1 in metastatic breast cancer [14], p70S6K1 in colon cancer [15], c-Myc in non-small cell lung cancer [16] and the transcription factor STAT1 in colon cancer [17]. MiR-145 is also known to regulate OCT4, SOX2, KLF4 and repress pluripotency in human embryonic stem cells [18]. Additionally, a very recent study showed that miR-145 directly targets the insulin-like growth factor receptor I (IGFR-1) in human bladder cancer cells [19].

The present study provides important insights into the tumor suppressor role of miR-145 in a well-known tumor-promoting network that includes MUC13. The study delineates the association of alterations in miR-145 levels with MUC13 and its potential role in PDAC initiation and progression. The results demonstrate that miR-145-induced downregulation of MUC13 is associated with slower growth of PanCa cell lines, gemcitabine chemo-sensitivity and tumor growth reduction in pancreatic xenograft mice model.

## RESULTS

### miR-145 is a post-transcriptional repressor of MUC13

**Fig.1:**
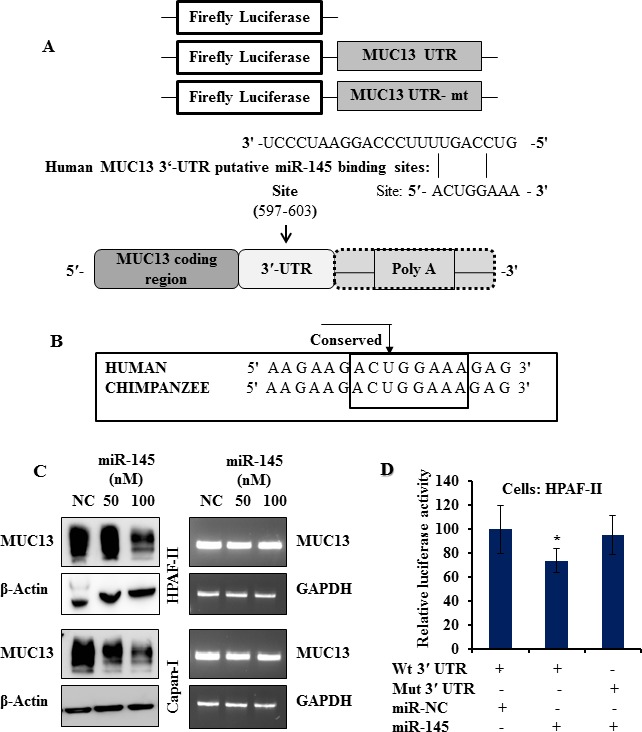
miR-145 negatively regulates the expression of MUC13 (A) Identification of a putative miR-145-binding site in the MUC13 3′ UTR region. Seven bases (597 through 603) of the MUC13 3′ UTR are perfect matches (seed sequence) for miR-145 binding. (B) Comparison of the MUC13-binding element among mammals demonstrates a high degree of conservation. (C) MUC13 expression on miR-145 transfection was examined at protein and mRNA levels by Western blot analyses and semi-quantitative reverse transcription–PCR (RT-PCR), respectively. (D) Luciferase reporter assay was used to examine the miR-145-mediated regulation of gene expression. HPAF-II cells were transiently co-transfected for 48 h with reporter plasmids (0.5 μg, WT or MUT) and 100 nM of miR-145 or NC mimic using Lipofectamine 2000. Luciferase (Firefly; test and Renilla, transfection efficiency control) activity was assessed using a dual-luciferase assay system. Data are presented as normalized fold change in luciferase activity (mean ± SD; n= 3, ^*^P <0.05).

*In silico* analysis through TargetScan, an online computational algorithm (http://www.targetscan.org/), revealed a putative 7-mer-1A binding site for miR-145 in the 3′ UTR of the *MUC13* transcript which is highly conserved across several mammalian species (Fig. 1 A, B). This suggested that miR-145 has an ability to target MUC13. We experimentally tested this in HPAF-II and Capan-1 cells (which express high levels of MUC13) *via* transient transfection of miR-145 mimic or non-targeting control mimic (NC). We observed a several fold increase in the miR-145 levels following transient transfection through qRT-PCR (Fig. S1A). Our data revealed a significant dose dependent downregulation of MUC13 at the protein level but no apparent change at the transcript level in miR-145 mimic transfected cells (Fig. 1C). This data suggests that miR-145 downregulates MUC13 expression through a post-transcriptional mechanism.

### miR-145 directly binds to the 3′ UTR of human MUC13

We employed luciferase assay to determine whether miR-145 targets the 3’ UTR of *MUC13* mRNA, as indicated by the TargetScan. We co-transfected the HPAF-II cells with miR-145 or NC and a firefly luciferase reporter plasmid containing a region of full-length 3′ UTR of *MUC13* mRNA harboring the miR-145 target site (position 597–603). As a control, MUC13 3′ UTR mutated vector was constructed and the specific sites targeted by the microRNAs were deleted.

The luciferase activity was substantially decreased (by 25%) in cells transfected with miR-145 as compared to NC transfectants. Cells transfected with MUT 3′ UTR were resistant to the suppressor activity of miR-145 (Fig. 1D), suggesting that miR-145 negatively regulates the expression of MUC13 by directly targeting 3′ UTR of the MUC13 transcript. The pmirGLO vector expressing Renilla luciferase was co-transfected into cells to normalize the transfection efficiency.

### miR-145 suppresses proliferation and invasion of PanCa cells

**Fig.2:**
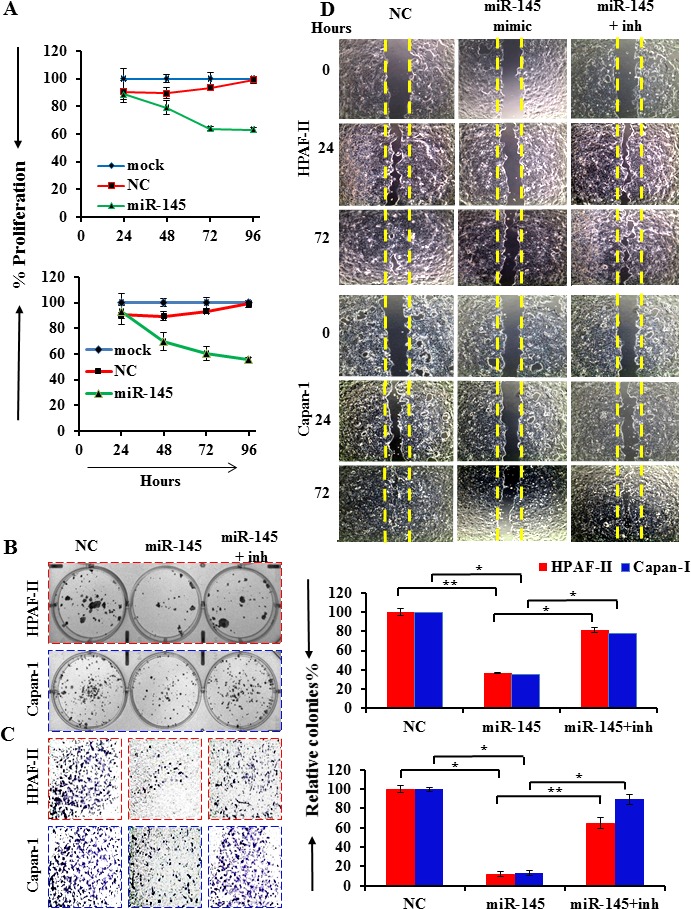
miR-145 inhibits growth, clonogenicity and invasion of PanCa cells Cells were transfected with either mock, miR-145 mimic or NC for 48 h. (A) Growth was monitored by MTS assay every day for the next 5 days and shown as percentage. (B and C) Transfected cells were analyzed for the ability to form colonies through clonogenicity assay. Data are presented as percent inhibition of clonogenic ability of miR-145 transfected cells as compared with their respective controls. Cells were also analyzed for invasion using matrigel invasion assay. Cells were photographed and counted using an imaging system. Bars represent mean ± SD; (n=3); *p<0.01 and **p<0.001. (D) Wound healing assay. The initial (0 h) and the residual gap length, 24/72 h after wounding, were analyzed from photomicrographs.

The effect of miR-145 on cell growth and metastasis was studied in PanCa cells, HPAF-II and Capan-1 that possess high constitutive expression of MUC13 and lost expression of miR-145. Therefore, we performed gain-of-function studies in these cells using miR-145 transfection to investigate its functional role in PanCa progression. Significant decrease in cell proliferation upon miR-145 transfection was observed through MTS assay as compared to the controls in HPAF-II and Capan-1 cell lines (96 h; HPAF-II: miR-145: 62.6±1.6, NC: 100.7±0.3, mock: 100±0.6, P<0.0001; and Capan-1: miR-145: 55.4±0.6, NC: 100±1.5, mock: 100±0.9, P<0.0005) (Fig. 2A). Another interesting observation is that miR-145 suppressed the clonogenic potential of HPAF-II and Capan-1 cells (70%), as determined by colony formation assays (Fig. 2B). Additionally, the effect of miR-145 on the invasiveness of cells was analyzed by matrigel invasion assay, following the migration of tumor cells under chemotactic drive in a Boyden's chamber. We observed a marked inhibition of cell invasion upon miR-145 transfection (48 h; HPAF-II: miR-145: 90.0±0.5, miR-145+inh: 30.1±3.4, NC: 0.0±2.4, P<0.01; and Capan-1: miR-145: 91.5±0.5, miR-145+inh: 10.3±4.0, NC: 0.0±2.7, P<0.01; Fig. 2C). Further, the miR-145 induced inhibition of cell migration was also performed through scratch assay. We observed a marked decreased cellular migration in the miR-145-transfected cells, HPAF-II (3-fold), and Capan-1 (3-fold) as compared with their respective controls at 24, 72 h (Fig. 2D) and 48 h (Fig. S1B). The inhibitor of miR-145 abrogated the effects of miR-145 in these cells. These data strongly suggest a role of miR-145 in PanCa progression.

### miR-145 regulates MUC13 associated key oncogenes

**Fig.3:**
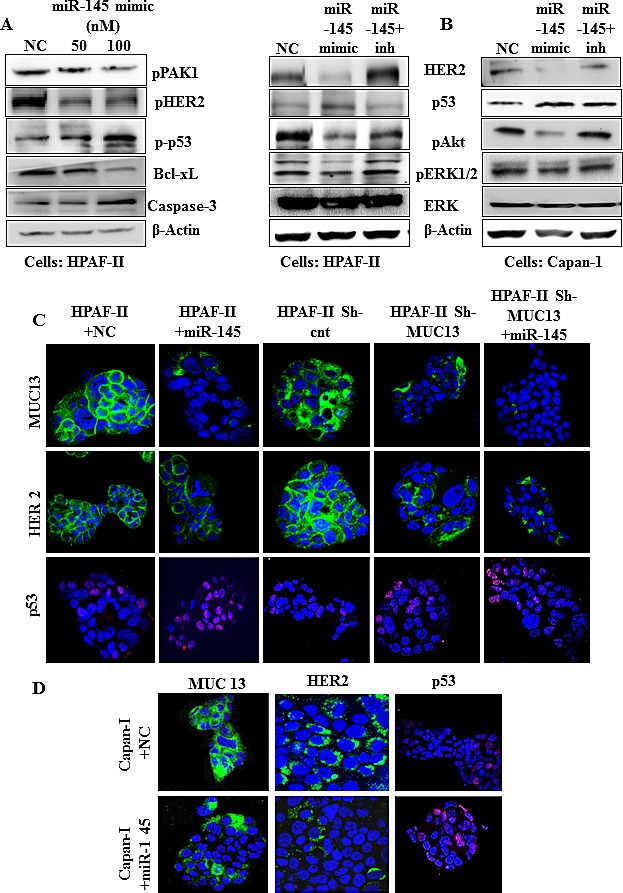
miR-145 inhibits MUC13 and its associated proteins in PanCa cells (A and B) Cells were transfected with miR-145 mimic, NC or miR-145 inhibitor in addition to miR-145 mimic for 48 h. Immunoblotting was performed for analysis of indicated proteins. (C and D) Confocal microscopy of HPAF-II, Capan-1 and HPAF-II sh-MUC13 cells treated with NC and miR-145 mimic. Data show a decrease in MUC13 (green) and HER2 (green) and an increase in p53 (pink) levels that reciprocated the results from the RNAi experiments using HPAF-II sh-MUC13 cells.

MUC13 expression enhances tumorigenic cell signaling pathways and is known to stabilize and increase HER2 and its downstream targets in PanCa [10]. We studied the biological function and significance of the regulation of MUC13 protein levels by miR-145 in PanCa cells. The MUC13+ cells (HPAF-II and Capan-1) were transfected with miR-145 or NC. The overexpression of miR-145 resulted in the potent inhibition of MUC13 protein and its related targets, *HER2, pAKT* and PAK1 [10], whereas p53 expression was increased (Fig. 3A). The cells treated with miR-145, in the presence of miR-145 inhibitor, had no effect on the basal protein levels (Fig. 3B). This confirmed that the molecular alterations observed were due to miR-145 restoration. Additionally, decreased MUC13 expression by miR-145 and its effect on the related targets, HER2 and p53, were also confirmed by immunofluorescence using confocal microscopy. These results corroborated shRNA experiments where similar effects with HPAF-II sh-MUC13 were observed (Fig. 3C and D). These results strongly suggest that miR-145 negatively regulates MUC13 protein expression and its associated targets.

### miR-145 increases gemcitabine sensitivity in PDAC cells

**Fig.4:**
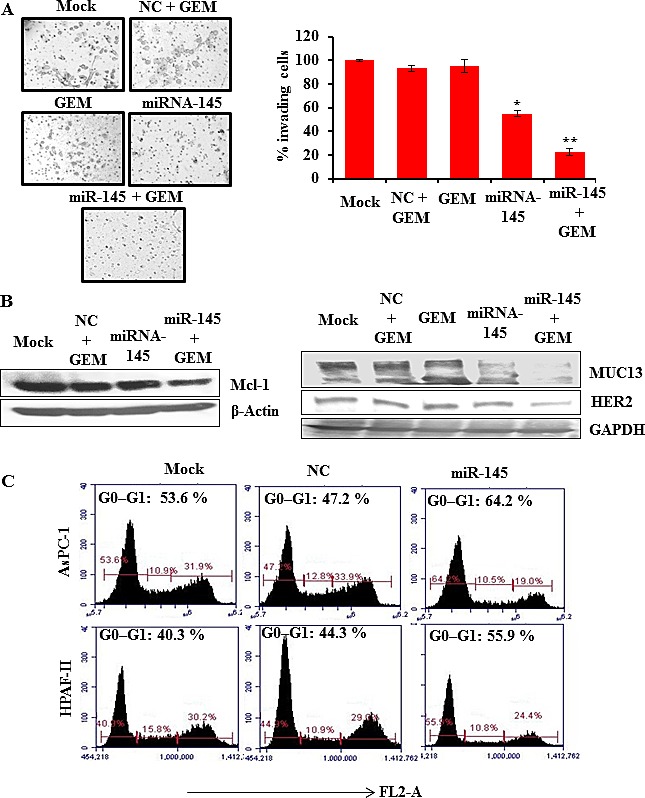
miR-145 increases gemcitabine sensitivity in PanCa cells AsPC-1 cells (gemcitabine resistant cells) were transfected with miR-145 mimic or NC and then treated with a gemcitabine-conditioned medium (100 nM) for 48 h followed by the (A) matrigel invasion assay. Cells were photographed and counted using an imaging system. Bars represent mean ± SD; (n=3); *p<0.01 and **p<0.001. (B) Western blotting for the analysis of expression of HER2, MUC13 and the gemcitabine target, Mcl-1. (C) Flow cytometry analysis of miR-145-transfected human pancreatic ASPC-1 and HPAF-II cells indicating an increase in the G0–G1 stage. Data are representative of one of three similar experiments.

The current standard care for metastatic pancreatic cancer is gemcitabine but success is poor due to emergence of drug resistance [20]. To assess whether miR-145 affects gemcitabine sensitivity in PDAC cells, we first determined the miRNA-145 expression in PanCa resistant cell lines. miR-145 was found to be differentially expressed between normal and PanCa cells, including gemcitabine resistant cells, as detected by qRT-PCR (Fig. S2B). This suggested that miR-145 might affect gemcitabine sensitivity in PanCa cells. To test this, gemcitabine resistant AsPC-1 cells were transiently transfected with miR-145 and then treated with a gemcitabine-conditioned medium (100 nM) for 48 h. The matrigel invasion assay showed that miR-145 decreased the number of invading cells and that gemcitabine showed enhanced effects under miR-145 restoration, clearly suggesting that miR-145 increases gemcitabine sensitivity to inhibit PanCa cell invasion. We also investigated the effect of gemcitabine on protein expression of HER2, MUC13 and the gemcitabine target Mcl-1 expression after miR-145 transfection through Western blotting. We found a significant effect on the inhibition of these proteins (Fig. 4B).

### miR-145 promotes cell death in PanCa cells

To determine the effect of miR-145 on cell cycle, MUC13 expressing HPAF-II and AsPC-1 cells were transfected with miR-145. Real-time PCR analysis showed 6-fold expression in miR-145 expression at 48 h after transfection over control (Data not shown). Cell cycle analyses using flow cytometry showed a concomitant increase in the G0–G1 stage with a simultaneous decrease in G2–M phase when AsPC-1 cells were transfected with the precursor of miR-145 compared to control cells (Fig. 4C).

### miR-145 inhibits tumor growth *in vivo*

**Fig.5:**
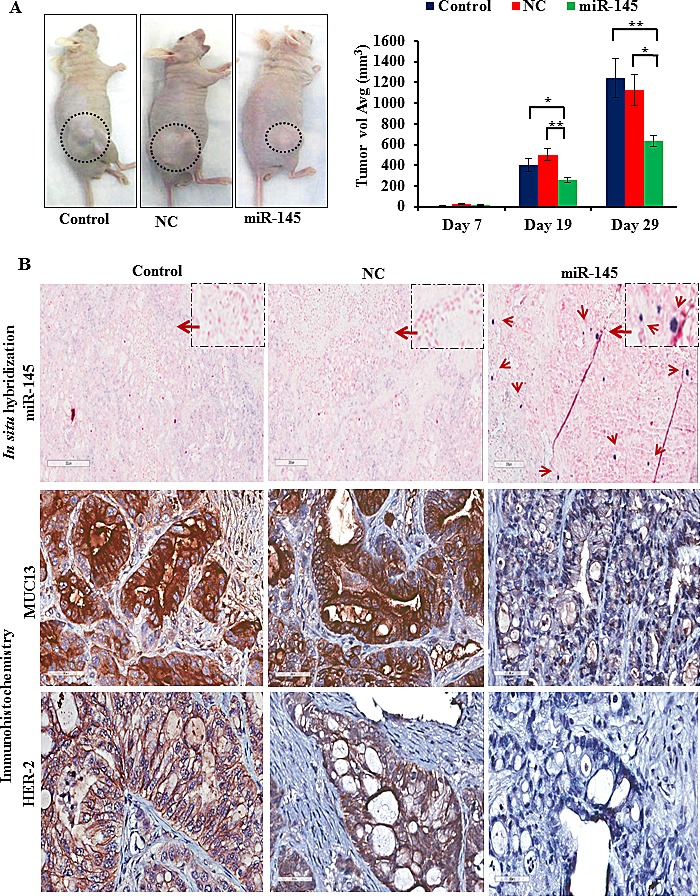
miR-145 inhibits tumor growth *in vivo* The antitumor effect of miR-145 was confirmed by *in vivo* experiments using xenograft models. (A) The antitumor effect of miR-145 was analyzed after intratumoral administration of miR-145 in established HPAF-II tumors. Average tumor volumes were calculated. Bars represent mean ± SD; *p<0.05 and **p<0.01. (B) Also, the xenograft tumors from miR-145 treated mice were analyzed for changes in MUC13 and HER2 expression (Original magnifications 40X) through IHC and miR-145 levels (Original magnifications 10X) using *in situ* hybridization followed by microscopy.

The antitumor effect of miR-145 was confirmed by *in vivo* experiments using PanCa xenograft mouse model. To investigate the antitumor effect, intratumoral miR-145 was administered in established HPAF-II tumors. The tumor volume regressed drastically with miR-145 replenishment compared to control and NC treated mice that showed gradual increase in tumor volume over time (Fig. 5A and S1C). Differences occurred at about day 19. The values at each time point were examined by comparing the two control conditions, control and NC (Cs) vs mir-145 (these analyses are termed ‘Time xx- A vs Cs’) Table S1. Differences were not significant for time points 7, 11, and 15. For time points 19, 22, 26, and 29, mir-145 was significantly different than the control conditions, and was lower. The values for mir-145 were examined for this condition alone, by comparing the time 7 value to others (‘time 7 vs miR-145). Here again we see that the difference appears at the day 19 point.

The tumor tissue samples were further analyzed for the expression of miR-145 levels, MUC13 and HER2 by *in situ* hybridization (ISH) and immunohistochemistry (IHC). We observed severe inhibition of MUC13 and HER2 levels in mice injected with miR-145 (Fig. 5B).

### miR-145 expression is clinically correlated with MUC13 expression

**Fig.6:**
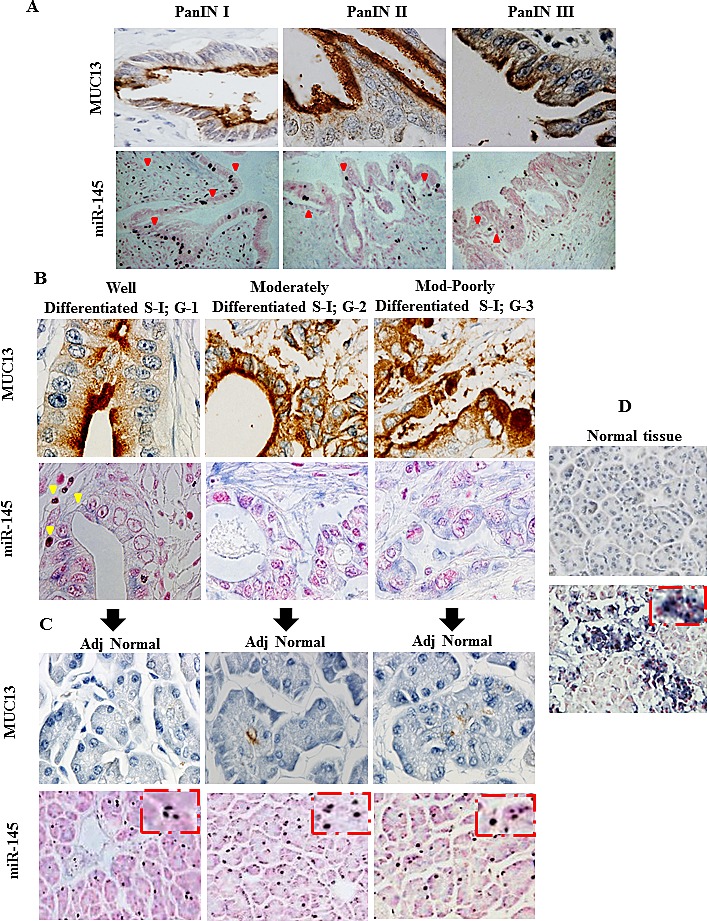
MUC13 expression is correlated with miR-145 expression in clinical samples Immunohistochemistry and *in situ* hybridization was used to detect MUC13 and miR-145, respectively, on the tissue microarray slides (procured from US Biomax, Inc., Rockville, MD) in various (A) PanIN lesions (original magnifications: MUC13 60X; miR-145 20X), (B) adenocarcinoma (original magnifications 60X) and (C) adjacent normal (Adj) (original magnifications: MUC13 40X; miR-145 20X) and normal pancreatic cancer cells (original magnifications: 20X).

To determine a correlation of MUC13 expression with miR-145 expression, human tumors and their adjacent normal tissues were used for MUC13 IHC and miR-145 ISH (Fig. 6). This revealed a strong correlation between MUC13 and miR-145 in both tumor and normal tissues. Our study demonstrates no or faint expression of MUC13 in human normal pancreatic tissues while having high miR-145 expression. Early pancreatic intraepithelial neoplasia (PanIN I) has shown moderate levels of MUC13 expression that is predominantly localized at the apical cell membrane and is lower in the cytoplasm, but shows higher miR-145 expression. But as the PanINs progressed to PanIN II and III, an increasing MUC13 expression was observed. In the later stages, MUC13 was found to be aberrantly localized with significantly high expression in the cytoplasm and nucleus among the increasing grades of PanCa (well, moderately, and poorly differentiated) tissue samples. The levels of miR-145 were observed to be very low or absent in late stage pancreatic intraepithelial neoplasia (PanIN II and III) and at later stages of PanCa. However, in adjacent normal tissues, MUC13 was localized at the apical membrane, and its expression was very low while miR-145 was found to be very high. These observations suggest that the consistent decrease in miR-145 expression and subsequent higher expression of MUC13 may play a role in the development and progression of PanCa.

## DISCUSSION

Understanding of aberrant regulatory mechanisms involved in pancreatic cancer progression is of utmost importance. An intensive focus is required to identify approaches that can fine-tune the expression of genes that are involved in disease progression and management [21, 22]. Mucins have been identified as potential tumor markers and are attractive therapeutic targets [23-26]. MUC13 is highly upregulated in PanCa and is involved in pancreatic tumorigenesis [10]. Therefore, investigating the regulation of MUC13 can reveal promising therapeutic approaches for PanCa treatment. The results obtained in this study, for the first time, provide information regarding the regulation of MUC13 expression in PanCa. We have identified that miR-145 regulates MUC13 expression in PanCa. Disruption of microRNAs has important implications in the etiology, treatment and pathogenesis of PanCa [2, 3]. Our investigations revealed that miR-145 is inversely correlated to MUC13 expression in PanCa cell lines (Fig. S2B) and pancreatic tumor tissues (Fig. 6). Thus, in the present study, we sought to delineate the association of alterations in miR-145 levels with MUC13 and its role in PanCa initiation and progression. This study elucidates the role of miR-145 as a novel regulator of MUC13, a finding that has not previously been reported.

We identified two miRNAs, miR-145 and miR-132 as MUC13 suppressing miRNAs (Fig. S2A). However, in the current study, we have focused on investigating the functional aspects of miR-145 in pancreatic pathogenesis. We have demonstrated that miR-145 regulates MUC13 expression in PanCa by directly targeting its 3′ UTR (Fig. 1C). We also observed that ectopic expression of miR-145 in MUC13-expressing HPAF-II cell lines resulted in MUC13 down regulation at the protein level. It inhibits MUC13 related known key oncogenes, HER2 and increases levels of the tumor suppressor p53 (Fig. 3). We have previously shown that MUC13 promotes PanCa invasion and metastasis [10]. Our study provides compelling evidence that expression of miR-145 causes suppression of tumor growth through the inhibition of MUC13 expression. These results indicated that miR-145 is an important tumor suppressor miRNA in PanCa and its replenishment in PanCa cells effectively inhibits their tumorigenic phenotypes (Fig. 2). The use of miR-145 inhibitor confirmed that the cellular and molecular alterations observed were indeed due to miR-145 restoration. Additionally, the RNAi experiments using HPAF-II sh-MUC13^−/−^, reciprocated the observed effects that were seen through miR-145 transfection. This clearly suggests that miR-145 silences the MUC13 expression and regulates the expression of its critical tumor target genes that are involved in pancreatic pathogenesis.

Additionally, it was intriguing that gemcitabine treatment resulted in inhibition of cell invasion in AsPC-1 cells that were transfected with miR-145. This approach showed an enhanced inhibitory effect on MUC13, HER2 and gemcitabine target, Mcl-1, expression. Furthermore, *in vivo* studies demonstrated a tremendous reduction in tumor growth in HPAF-II xenograft mice that were injected intratumorally with miR-145. Moreover, IHC analyses of tumor tissues revealed an inhibition of MUC13 expression in miR-145 treated tumors. Interestingly, experimental observation revealed a tremendous reduction in HER2 levels, which is aberrantly expressed in PanCa [27] (Fig. 5). Thus, this suggests that miR-145 inhibits MUC13 and its related targets and leads to tumor regression in xenograft mice. This data suggests that miR-145, besides inhibiting MUC13 and pancreatic tumorigenesis, also sensitizes PanCa cells to gemcitabine treatment.

Additionally, we have presented evidence for an inverse correlation of miR-145 and MUC13 expression in human PanCa clinical specimens. We observed that MUC13 is markedly expressed as early as in PanIN lesions and progresses from PanIN I to PanIN III and its expression is inversely correlated to miR-145 expression (Fig. 6). A very strong expression of miR-145 was observed in normal tissues adjacent to the cancerous tissues as well as in normal pancreatic tissues with less or no MUC13 expression (Fig. 6C).

These observations suggest that the consistent decrease in miR-145 levels may play a role in the development and progression of PanCa. In conclusion, this finding suggests that miR-145 is a tumor suppressor in PanCa. It can be a successful therapeutic strategy for cancer through its inhibitory effects on MUC13. Our study provides important insights into the role of miR-145 in a well-known tumor-promoting network that involves MUC13, which may provide a route to therapeutic miRNA intervention in PanCa.

## MATERIALS AND METHODS

### Cell culture

All pancreatic cancer (PanCa) cell lines were purchased from the American Type Cell Culture (ATCC) collection and maintained at 37^0^C in a recommended growth medium (Capan-I: RPMI and HPAF-II: DMEM/Ham's F12), supplemented with 10% FBS and antibiotics (Hyclone Laboratories). HPAF-II cells were transduced with five different constructs of MUC13 specific shRNA lentiviral particles (Sigma) according to the manufacturer's protocol [10]. Stable cells were then selected and maintained in the media containing 3mg/ml puromycin (Sigma). The wild-type and vector control cells did not show any significant differences.

### Transfection procedure

PanCa cells, HPAF-II, Capan-I and AsPC-1 cells were transiently transfected with *mirVana* miR-145 mimics (MC11480; Applied Biosystems), miR-132 mimics (Assay id MC10166; Applied Biosystems) or non-targeting control mimic (NC) (catalog number AM17111; Applied Biosystems). Mock transfection containing only the transfection reagent was used as a control. The cells were transfected using Lipofectamine 2000 (catalog number 11668-019; Invitrogen) following the manufacturer's protocol. Cells were pelleted after 48-72 h of transfection for flow cytometry, RNA and protein extraction.

### Dual-luciferase 3′ UTR-reporter assay

Dual-luciferase 3′ UTR reporter assay was carried out to validate MUC13 as a direct target of miR-145. The luciferase-UTR reporter constructs were generated by inserting the MUC13 3′ UTR carrying a putative miR-145 binding site into pmirGLO control vector (catalog number E1330; Promega). Additionally, in the wild type MUC13 3′ UTR (WT-MUC13 3′ UTR), a mutant MUC13 3′ UTR (MUT-MUC13 3′ UTR) reporter construct was made by site-directed mutagenesis in the putative target site of miR-145 using Quickchange XL site-directed mutagenesis kit (Agilent Technologies, Santa Clara, CA). All PCR products were verified by DNA sequencing. HPAF-II cells were transiently co-transfected with reporter plasmids (1μg) and 100 nM of miR-145 or miR-NC for 48 h. Luciferase assays were performed using a luciferase assay kit (catalog number E2940; Promega) according to the manufacturer's protocol. The normalized luciferase activity was expressed as a ratio of firefly luciferase to Renilla luciferase units.

### Reverse transcription–quantitative real-time polymerase chain reaction

Total RNA was extracted using TRIzol reagent (catalog number AM 9738; Invitrogen). The integrity of the RNA was checked with an RNA 6000 Nano Assay kit and 2100 Bioanalyzer (Agilent Technologies, Santa Clara, CA, USA). For miRNA detection, 100 ng total RNA was reverse transcribed into cDNA using specific primers designed for miRNA analysis (Assay id 002278; Applied Biosystems) using High Capacity cDNA Reverse Transcription kit (catalog number 4368814; Applied Biosystems). The mature miRNA and *MUC13* expression levels were determined by real-time PCR using Taqman PCR master mixture and specific primers using Taqman expression Assay (Assay id 002278, Hs01550533_m1 respectively; Applied Biosystems). The expression of miRNA was normalized with the expression of U6 snRNA (Assay id 001093; Applied Biosystems) and 18s (catalog number 4319413E, Applied Biosystems). Normal pancreatic cells, HPNE were used as calibrator control. The expression of *MUC13* was normalized to GAPDH gene.

A semi-quantitative PCR was performed to amplify MUC13 according to the standard three-step procedure. MUC13 mRNA was detected using SYBR green with primers specific to MUC13 and glyceraldehyde-3-phosphosphate dehydrogenase (GAPDH) specific [28]. Average levels GAPDH were used as an internal control [29].

### Cell proliferation and clonogenicity assays

Cell proliferation assays were carried out according to the standard methods previously described [30]. After transfection of cells, viability was determined at 24, 48, 72 and 96 h using MTS ([3-(4,5-dimethylthiazol-2-yl)-5-(3-carboxymethoxyphenyl)-2-(4-sulfophenyl)-2H-tetrazolium] reagent (catalog number G5421, Promega) according to the manufacturer's protocol and absorbance was measured at 490 nm. For the anchorage-dependent colony forming assay, cells (5 × 10^2^) were transfected and incubated for 10 days, fixed with 100% methanol and stained with hematoxylin. The colonies (>50 cells) were counted manually and plotted as described earlier [30, 31].

### Cell cycle analysis

Cell cycle analysis was performed 48 h after transfection using Propidium iodide (catalog number P-4170, Sigma) and analyzed by Accuri flowcytometer.

### Migration and Invasion assay

The wound healing migration assay was used to evaluate the effect of miR-145 transfection on the migratory ability of cancer cells [32]. Cells were transfected with miR-145 mimics, NC in presense or absence of miR-145 inhibitor (Assay id MH11480; Applied Biosystems). Transfected cells were plated and the cell monolayer was scraped using a micropipette tip. The initial (0 h) and the residual gap length of 48 to 72 h after wounding were calculated from photomicrographs. Additionally, the effect of miR-145 transfection on invasiveness was determined by using matrigel chambers (catalog number 734-1048, BD Biosciences), as discussed previously [10].

### Western blot

Cells were transfected with miR-145 mimics, NC in presence or absence of miR-145 inhibitor (Assay id MH11480; Applied Biosystems) and total protein was extracted from PanCa cells, followed by Western blotting as previously described [30, 33]. Proteins were analyzed by immunoblotting with anti-MUC13 MAb (clone PPZ020), anti-HER2 (catalog number A0485; DAKO), anti-phospho-HER2 (tyr1248) (catalog number 2247; Cell Signaling), anti-phospho-PAK1 (catalog number 2605; Cell Signaling), anti-p44/42 MAPK (ERK1/2) (catalog number 9102; Cell Signaling), PAK1 (catalog number 2602; Cell Signaling), anti-phospho-p44/42 MAPK (ERK1/2) (catalog number 9101; Cell Signaling), anti-AKT (catalog number 9272; Cell Signaling), anti-phospho-AKT (Thr308) (catalog number 2965; Cell Signaling), anti-p53 (catalog number 2527; Cell Signaling), anti caspase-3 (catalog number 9662; Cell Signaling), anti-Bcl-xL (catalog number 2764; Cell Signaling), anti Mcl-1 (catalog number 5453; Cell Signaling), GAPDH (catalog number 5174; Cell Signaling) and anti-β–Actin (Sigma).

### Immunofluorescence and confocal microscopy

Immunofluorescence staining was performed to determine the effect of miR-145 transfection on the protein level of MUC13 and other related key oncogenic proteins. Cells were grown at a low density for 24h on the chamber slides (Nalge Nunc Int) and processed for immunofluorescence as described [28]. Cells were incubated with anti-MUC13 MAb (clone PPZ020) or anti-HER2 (catalog number A0485; DAKO), anti-p53 (catalog number 2527; Cell Signaling) followed by an incubation with species specific Alexa Fluor 488 (catalog number A11029; Invitrogen) or Alexa Fluor 568 (catalog number A11036; Invitrogen) secondary antibodies and were mounted in Fluoro Care Anti-Fade mounting medium (BioCare Medical, CA, USA). The cells were examined under a laser confocal microscope (Nikon Corporation).

### Immunohistochemistry

Immunohistochemistry was used to detect MUC13 in the tissue microarray slides (procured from US Biomax, Inc.). Also, the xenograft tumors from miR-145 treated mice were analyzed for changes in MUC13 and HER2 expression. The slides were stained with anti-MUC13 MAb and HER2 using Biocare's MACH4 Universal HRP-Polymer kit (Biocare Medical, CA, USA). Expression of anti-MUC13 and anti HER2 was determined as previously described [28].

### In situ hybridization for miRNAs

Inorder to detect the expression of miR-145 in FFPE tissues of control and treated xenograft mice, *in situ* hybridization technique was used using Biochain kit (catalog number K2191050; Biochain IsHyb In Situ hybridization kit). Briefly, tissues were deparaffinized and fixed in 4% paraformaldehyde in DEPC-PBS for 20 min. They were subjected to digestion using 2X standard saline citrate and 0.1% Triton-X for next 25 min. The tissue were prehybridized with prehydridization solution provided with the kit for 4 hours at 48^o^C. This followed the hybridization of the slides with hybridization buffer and digoxigenin labelled probe (EXIQON, Woburm, MA, USA) at 45^o^C overnight. After stringent washing of tissue slides with various grades of standard saline citrate, the slides were blocked using 1X blocking solution provided with the kit. This followed the subsequent incubation of tissues overnight with the AP-conjugated anti-digoxingenin antibody. Further, the slides were washed for 5 min with 1X Alkaline Phosphatase buffer twice. The final visualisation was carried out with NBT/BCIP (Pierce, Rockford, IL, USA) followed by nuclear fast red counterstaining. The experiment was conducted according to the manufacturer's protocol (Biochain and EXIQON). The slides were mounted and analyzed under microscope.

### *In vivo* tumorigenic assay

Six-week-old athymic Nu/nu nude mice were purchased from Charles River Laboratories International, Inc. (Wilmington, MA), and maintained in a pathogen-free environment. Three control, six NC and six miR-145 mice groups were used (total of 15 mice). In brief, MUC13 expressing PanCa HPAF-II cells were suspended in PBS and Matrigel (BD Bioscience, San Jose, CA) at a 1:1 ratio. Cell suspension (5 × 10^6^ cells) was injected subcutaneously into the right flank of each xenograft nude mice. Once palpable tumors developed (average volume 80 mm^3^), mice tumors were treated with miR-145 injections nine times. 100 nM synthetic miR-145 complexed with 100 μl Invivofectamine 2.0 transfection reagent (catalog number 1377501; Ambion, Austin, TX) in 50 μl PBS was delivered four times intratumorally every alternate day. Then, 100 nM synthetic miR-145 complexed with 2 μl siPORT Amine transfection reagent (catalog number AM4502; Ambion, Austin, TX) [34] in 50 μl PBS was delivered for next five times intratumorally every alternate day. Tumor growth was followed for 21 days from first injection until tumors reached 700 mm^3^ total volumes, at which time mice were euthanized. The organs, including pancreas, were harvested and checked for metastases. Tumor volume (V) was estimated from the length (l), width (w), and height (h) of the tumor using the formula V ¼ 0.52(l × w × h), as described previously [28]. Tumor volumes (V) were examined as a function of time (discrete), group (control, NC, miR-145), and interaction between them. Primary analyses involved the comparison (for each time point separately) between control and NC vs mir-145, performed as planned comparisons. All animal care was in accordance with institutional guidelines and all animal experiments were done using protocols approved by the Institutional Animal Care and Use Committee (IACUC).

### Statistical Analysis

Statistical significance of the data was performed by a Student's t test. Differences with P values of <0.05 were considered significant. The data from mouse studies were log-transformed for analysis. The basic analysis is a mixed-effect repeated measures (MERM) analysis using PROC GLIMMIX in SAS v9.3 (SAS Institute, 2012).

## SUPPLEMENTARY MATERIAL FIGURES AND TABLE


