# Supplementary material for: MicroRNA-145 targets MUC13 and suppresses growth and invasion of pancreatic cancer
Source: Oncotarget. 2014 Jul 30;5(17):7599–609. doi: 10.18632/oncotarget.2281 (PMC4202147; doi:10.18632/oncotarget.2281)
Supplement: Supplementary file 1 [file oncotarget-05-7599-s001.pdf]

MicroRNA-145 targets MUC13 and suppresses growth and invasion of pancreatic cancer

Supplementary Material

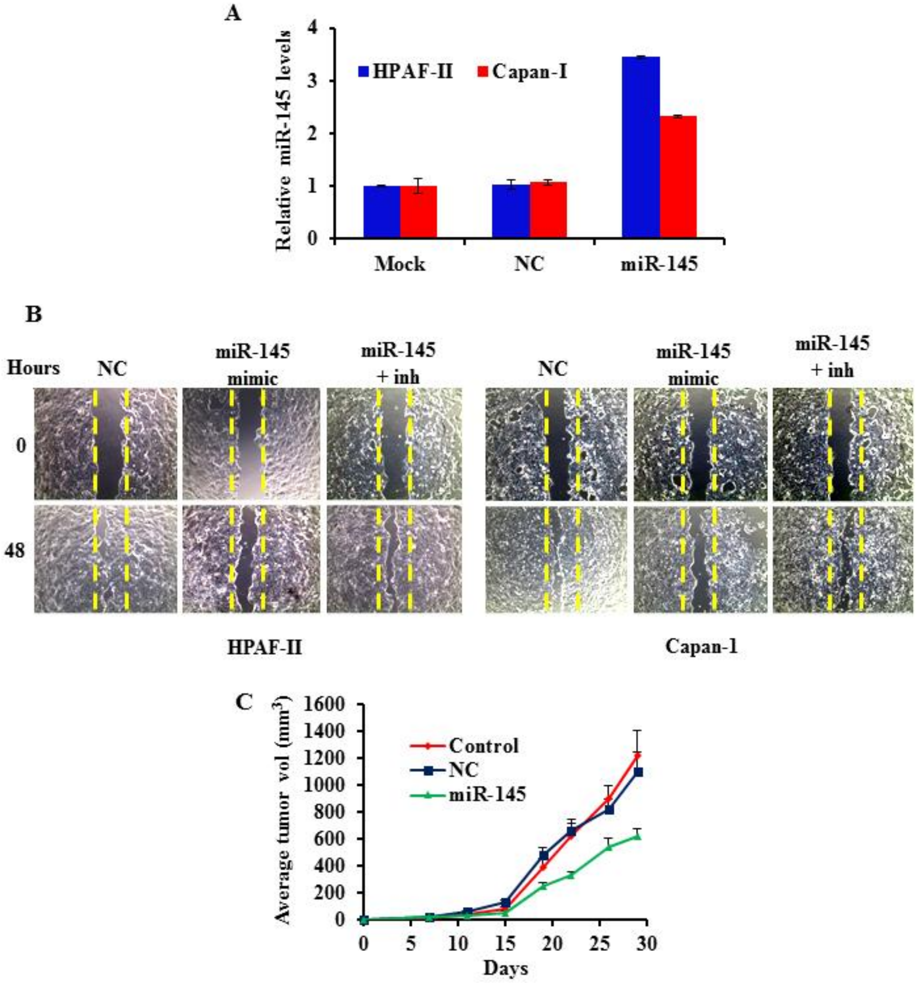

Fig. S1: miR-145 inhibits migrataion ability of PanCa cells and tumor growth in xenograft mice. (A) Following miR-145 transfection fold changes in the miR-145 levels were determined through qRT-PCR. U6 small nuclear RNA was used as an internal control for relative quantitation. **(B)** miR-145 transfection inhibits migration ability of HPAF-II and capan-1 cells at 48 h as seen through Wound healing assay. **(C)** The average tumor volumes in mice untreated or treated with miR-145 and NC intratumorally.

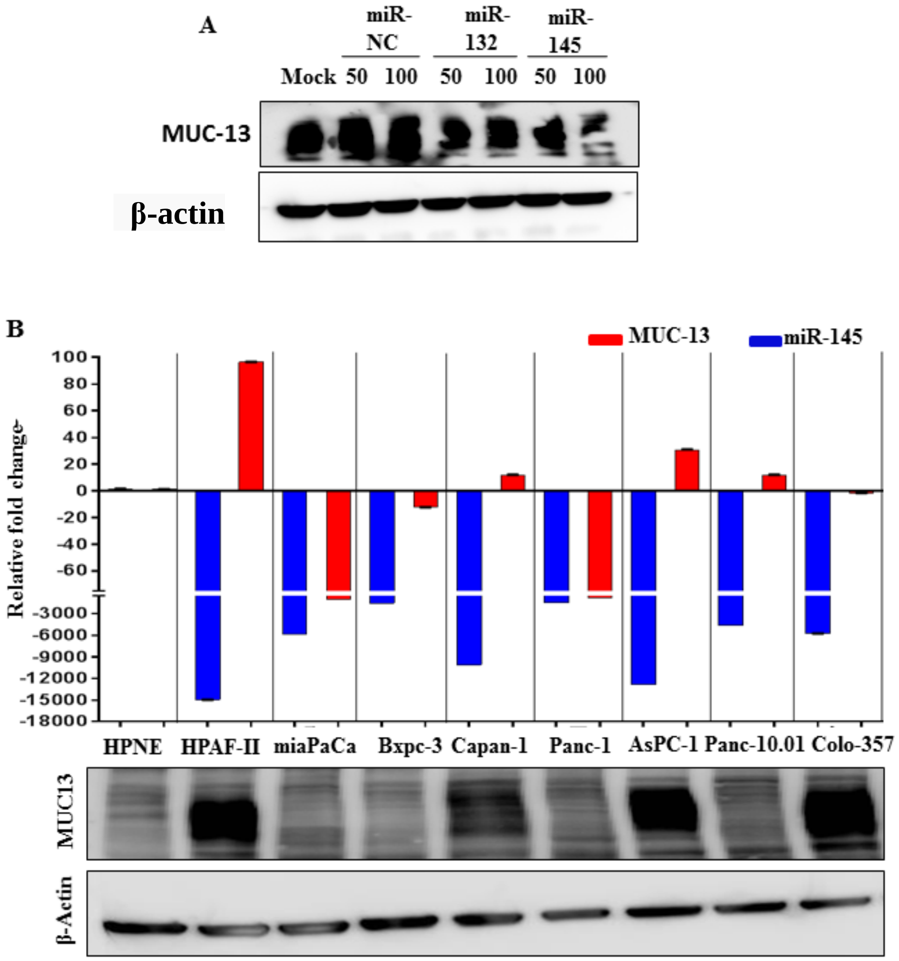

Fig. S2: miR-132 and miR-145 regulate MUC13 expression. (A) Following miR-132 and miR-145 transfection using lipofectamine 2000 in HPAF-II cells, the regulation of MUC13 expression was examined at protein level by Western blot analyses. β-Actin was used as internal control. **(B)** Inverse correlation of expression of miR-145 and MUC13 in PanCa. Expression of mature miR-145 and MUC13 was examined in normal and pancreatic cancer cells by qRT-PCR and Western blotting, respectively. U6 small nuclear RNA was used as an internal control for relative quantitation. Normal pancreatic cells, HPNE were used as calibrator control.

Table S1: Specific planned comparisons to test specific comparisons.

Contrast	Test F	Mean
Early v late - miR	22.08	<.0001
Time 7 v 15 - miR	0.45	0.5033
Time 7 v 19 - miR	3.80	0.0553
Time 7 v 22 - miR	4.65	0.0344
Time 7 v 26 - miR	6.34	0.0140
Time 7 v 29 - miR	6.89	0.0106
Time 7 - A vs Cs	0.02	0.8900
Time 11- A vs Cs	0.28	0.5975
Time 15- A vs Cs	1.79	0.1848
Time 19- A vs Cs	9.35	0.0031
Time 22- A vs Cs	16.23	0.0001
Time 26- A vs Cs	9.40	0.0031
Time 29- A vs Cs	17.47	<.0001

The values at each time point were examined by comparing the two control conditions, control and NC (Cs) vs mir-145 (these analyses are termed 'Time xx- A vs Cs'). miR: miR-145; Cs: controls.
